# Lessons learned from hemolytic uremic syndrome registries: recommendations for implementation

**DOI:** 10.1186/s13023-021-01871-9

**Published:** 2021-05-25

**Authors:** Mina Lazem, Abbas Sheikhtaheri, Nakysa Hooman

**Affiliations:** 1 Department of Health Information Management, School of Health Management and Information Sciences, Iran University of Medical Sciences, Tehran, Iran; 2 Health Management and Economics Research Center, Health Management Research Institute, Iran University of Medical Sciences, Tehran, Iran; 3 Pediatric Nephrology Department, Aliasghar Clinical Research Development Center (AACRDC), Aliasghar Children Hospital, Iran University of Medical Sciences, Tehran, Iran

**Keywords:** Registry, Hemolytic uremic syndrome, Thrombotic microangiopathy

## Abstract

**Background:**

Hemolytic uremic syndrome (HUS) is a rare condition which diagnosed with the triad of thrombocytopenia, microangiopathic hemolytic anemia, and acute renal injury. There is a high requirement for research to discover treatments. HUS registries can be used as an important information infrastructure. In this study, we identified and compared the different features of HUS registries to present a guide for the development and implementation of HUS registries.

**Results:**

The purposes of registries were classified as clinical (9 registries), research (7 registries), and epidemiological (5 registries), and only 3 registries pursued all three types of purposes. The data set included demographic data, medical and family history, para-clinical and diagnostic measures, treatment and pharmacological data, complications, and outcomes. The assessment strategies of data quality included monthly evaluation and data audit, the participation of physicians to collect data, editing and correcting data errors, increasing the rate of data completion, following guidelines and data quality training, using specific data quality indicators, and real-time evaluation of data at the time of data entry. 8 registries include atypical HUS patients, and 7 registries include all patients regardless of age. Only two registries focused on children. 4 registries apply prospective and 4 applied both prospective, and retrospective data collection. Finally, specialized hospitals were the main data source for these registries.

**Conclusion:**

Based on the findings, we suggested a learning framework for developing and implementing an HUS registry. This framework includes lessons learned and suggestions for HUS registry purposes, minimum data set, data quality assurance, data collection methods, inclusion and exclusion criteria as well as data sources. This framework can help researchers develop HUS registries.

**Supplementary Information:**

The online version contains supplementary material available at 10.1186/s13023-021-01871-9.

## Background

Hemolytic uremic syndrome (HUS) is a clinical condition characterized by thrombocytopenia, thrombotic microangiopathy (TMA), and acute kidney injury [1]. The typical infectious cause of HUS is *Escherichia coli* (*E. coli*), producing Shiga toxin (typical, or STEC-HUS). However, patients sometimes should be screened for genetic causes or underlying diseases [2]. Diarrhea, vomiting, renal injury symptoms, and extrarenal manifestations (20% of cases) such as seizures are symptoms of the disease [3]. Atypical HUS (aHUS) is also caused by the uncontrolled activation of complement factors (a part of the immune system) [4], and is very chronic and progressive and eventually leads to kidney (or other systemic organs) injuries [5]. A wide range of therapies was suggested from conservative management to plasma exchange (PEX), and using Eculizumab (Ecu) [6]. A recent systematic review reports that the annual incidence of aHUS for all ages is 0.23 to 1.9 per 1 million people and 0.26 to 0.75 for patients at the age of 20 years old or less [7]. HUS is one of the various forms of TMA [8], and with symptoms such as thrombocytopenia, nonimmune microangiopathic hemolytic anemia, microvascular occlusion, and acute kidney injury, it is in the group of diseases known as TMAs [9].

HUS is a rare condition [10] which its clinical pattern can occur in a wide range of clinical scenarios [3]. Rare diseases are characterized by the large number and broad diversity of disorders and symptoms that are often incurable [11] that reduce the quality of life and increase mortality [12, 13]. Therefore, due to variations in the course of rare diseases, low prevalence, and other complications, there is a large gap between basic research in this field and the need to discover new therapies and drugs for rare diseases [14].

The development of a disease registry can help provide research opportunities and solve issues related to scientific studies in this field. Disease registry programs are organized systems which collect uniform data and evaluate the outcomes of a specific disease in a pre-defined population [15]. Therefore, registries facilitate research, especially in the rare disease domain. These systems can be used for patients’ recruitment for clinical trials, support health care management, and improve patient care [16].

Implementation of Rare Disease Registries (RDRs) helps scientists understand the variations about this type of disease and provides the necessary information for designing clinical trials. The population of patients with a rare disease is often small and geographically dispersed [17]. In this regard, RDRs enhance the quality and efficiency of clinical studies by providing more samples [18]. In addition to increasing knowledge about rare diseases, RDRs are an important source of data on drug safety and efficacy for post-market surveillance [17, 19].

Due to the importance of this type of disease registry, many countries have implemented HUS registries, each of which has its features [4, 20]. Despite designing different HUS registries and considering that there is no standard guide in the world on how to develop these types of registries [21], we identified and compared the different features of HUS registries and their similarities and differences to learn lessons regarding the features of HUS registries and to develop a guideline to design and implement an HUS registry.

## Method

The features of HUS registries in countries were identified by descriptive and comparative methods. The RDRs can be usually described from different aspects [22], and due to the available information; we considered the following aspects:

1. Purposes: Registries can be developed for different purposes [19] which can range from monitoring, control, and management of a disease to identify disease course and outcomes, as well as to evaluate the safety and effectiveness of interventions and treatments [23].
2. Inclusion and exclusion criteria: The registry team should determine the inclusion criteria, and indeed the eligibility of patients for registration. On the other hand, exclusion criteria exclude individuals from entering the registry [22, 23].
3. Minimum Data Set (MDS): Another important feature of registries is determining the specified MDS, which includes a group of disease-related data elements to be collected [24].
4. Data sources which are the settings and organizations from where the information is collected [22].
5. Determining data collection method which may include interviewing patients, reviewing health records of the patients, or direct clinical observation [25].
6. Data quality control is one of the activities which should be continuously done to assure and increase data quality [26–28].
7. Registrars: One of the necessities to make sure the correct data entry is to determine the relevant registrars. Registrars collect data and enter it into the registry system during their work or dedicated work [29].

This study was conducted to determine the comprehensive dimensions of features considered in the present HUS registries to achieve a learning framework for the optimal and effective implementation of HUS registries.

### Selection of HUS registries

In December 2020, we first searched the recognized HUS and TMA registries on the European Orphanet website and the registry official websites. To consider publications related to these registries, we also searched electronic databases, including ISI Web of sciences, Embase, PubMed (Medline), Scopus, and Google search engine. The terms "Hemolytic uremic syndrome/thrombotic microangiopathy registry" and "hemolytic uremic syndrome/thrombotic microangiopathy database" were used. The articles which described the features of HUS registries were considered. To collect data regarding some of these registries, we corresponded with their managers.

We considered specific registries for HUS (typical or atypical) or TMA registries with HUS inclusion criteria. The other inclusion criterion was the availability of information regarding the aspects of interest. After reviewing the registry and article titles, and our inclusion criteria (if any), 41 registries were identified, of which only 14 were related to HUS or TMA. Among them, 10 registries were finally selected. Other 4 registries were excluded because we could not find related information or these registries were a participant of global aHUS registry [30]. Six of 10 selected registries were identified from Orphanet and the others from articles and websites. Three registries related to TMA [31–33] due to the inclusion of patients with HUS were also selected (Table 1).

**Table 1.** 10 selected HUS registries

Reg. codes	Name (References)	Starting year	Country	Current situation	Patient number
Reg.1	Oklahoma TTP-HUS Registry [34–36]	1989	US	NA	434 (to 2015)
Reg.2*	International Registry of recurrent and familial HUS/TTP [37–39]	1996	Italy	Active	1200 (to 2016)
Reg.3*	French Registry of aHUS in children [40]	2005	France	Active	375 (to 2013)
Reg.4*	Italian Registry of Hemolytic Uremic Syndrome [41, 42]	2005	Italy	Active	NA
Reg.5	International Registry and biorepository for TMA [31, 43]	2007	US	Inactive	NA
Reg.6*	TTP/TMA Registry [44]	2011	Australia	Active	NA
Reg.7	German STEC-HUS Registry [45, 46]	2011	Germany	NA	631(to 2012)
Reg.8*	Atypical Hemolytic-Uremic Syndrome Registry [30, 47]	2012	US	Active	852 (to 2019)
Reg.9*	Turkish pediatric aHUS Registry [20, 48]	2013	Turkey	Active	146 (to 2017)
Reg.10	TMA Registry of North America (TRNA) [33]	2013	US	NA	NA

*From Orphanet

aHUS: Atypical Hemolytic Uremic Syndrome; HUS: Hemolytic Uremic Syndrome; NA: Not Available information; Reg.: Registry; STEC-HUS: Shiga toxin-producing *E. coli* HUS; TMA: Thrombotic Microangiopathy; TTP: Thrombotic Thrombocytopenic Purpura

### Data collection and analysis

We reviewed the relevant articles and websites and also communicated with the managers of selected registries by email to collect information for the features of these registries. We developed comparative tables (Additional file 1) to thematically compare these features and identify similarities and differences, and make suggestions to implement these registries.

## Results

We finally selected 10 HUS registry programs (Table 1). In the following section, their support centers or supervisors are introduced. Furthermore, the details of these registries are provided in Additional file 1.

These registries are introduced as follows. We used these registry numbers to refer to the name of these registries in the following sections.

Registry 1: Oklahoma TTP-HUS Registry is one of the oldest local registry systems set up at the Oklahoma Blood Institute and under the supervision of the University of Oklahoma Health Sciences Center to register any patient for whom PEX is requested [35].

Registry 2: International Registry of recurrent and familial HUS/thrombotic thrombocytopenic purpura (TTP) is also a global disease registry system set up with Mario Negri Institute of Pharmacological Research's support and supervision at the Clinical Research Center for Rare Diseases in Italy [39].

Registry 3: French registry of aHUS in children was launched as a hospital clinical research program at the Laboratory of Biological Immunology at the European Hospital Georges Pompidou under the supervision and support of the Association for Information and Research on Genetic Renal Diseases, France [40].

Registry 4: Italian registry of HUS is a national registry in Italy that the National Institute of Health has implemented as part of the activities of the Italian Society for Pediatric Nephrology [41, 42].

Registry 5: International registry and biorepository for TMA was supported by Northwell Health Clinical Integration Network (New York state health service provider) for clinical research on diseases in TMA group such as HUS [31, 43].

Registry 6: TTP/TMAs registry was set up by the Department of Epidemiology and Preventive Medicine at Monash University in Australia to establish a high-quality clinical and specialized registry to support research [32].

Registry 7: German STEC-HUS registry, implemented by the German Society of Nephrology, is based on a combination of two research projects in Hamburg and Hanover with the association of an IT company. An English version of the registry is now available, enabling other European countries to register patients with HUS [46].

Registry 8: Atypical HUS registry is a global, multicenter registry system of patients with aHUS developed by the National Institute of Health (NIH). This system is the product of collaboration among universities around the world and the American Lexicon Pharmaceuticals [30].

Registry 9: Turkish pediatric aHUS registry is a national web-based registry system similar to (but not included in) the global aHUS registry, which the Faculty of Hacettepe of University has implemented to enroll children with aHUS in pediatric nephrology hospitals in Turkey [20].

Registry 10: TMA Registry of North America (TRNA) was set up to overcome the limitations of research about rare TMA in the United States and to identify these patients for study at four US university centers (Columbia, Duke, Alabama in Birmingham, and Pennsylvania). It was decided to launch this program at the meeting of the American Society for Apheresis (ASFA) in 2013 [49].

### Comparison of the registries

Table 2 indicates the similarities and differences among the selected registries.

**Table 2:** Comparing the features of selected HUS registries

		Reg.1 [34–36, 50]	Reg.2 [37–39]	Reg.3 [40]	Reg.4 [41, 42]	Reg.5 [31, 43]	Reg.6 [32, 44]
Geographical coverage	Regional	✓	–	–	–	–	–
	National	–	–	✓	✓	–	✓
	International	–	✓	–	–	✓	–
Purposes	Clinical	✓	✓	✓	✓	✓	✓
	Research-based	✓	✓	✓	–	✓	✓
	Epidemiological-based	–	–	–	✓	✓	✓
Data set	Demographics	✓	✓	–	✓	✓	✓
	Clinical history	✓	✓	–	✓	✓	✓
	Paraclinical measures	✓	✓	–	✓	✓	✓
	Treatment	✓	✓	–	✓	✓	✓
	Outcomes	✓	✓	–	✓	✓	✓
	Biological samples	–	✓	–	–	✓	–
	Patient evaluation (safety/drug efficacy)	–	–	–	–	–	–
Data quality assessment strategy	Completion of data	✓	–	–	–	–	–
	Physician’s participation in data quality control	–	–	–	–	–	–
	Following data quality guidelines	–	–	–	–	–	✓
	Using of data quality indicators	–	–	–	–	–	–
	Evaluating and auditing data quality	–	–	–	✓	–	✓
	Training courses on data quality	–	–	–	–	–	✓
	Data errors tracking	–	–	–	✓	–	–
	Real-time evaluation of data at the time of its entry	–	–	–	–	–	–
Inclusion criteria	Atypical HUS	✓	✓	✓	✓	✓	✓
	Typical UHS	–	–	–	✓	–	–
	Children	✓	✓	✓	✓	✓	✓
	All ages	✓	✓	✓	✓	–	✓
Exclusion criteria	Atypical HUS	–	–	–	*	–	–
	Typical UHS	✓	–	✓	–	–	–
	Adults	–	–	–	–	✓	–
Registrars	Physicians (nephrologists)	✓	–	✓	–	✓	✓
Data collection	Retrospective	✓	✓	–	✓	–	–
	Prospective	✓	✓	✓	✓	✓	–
Data sources	Healthcare centers	✓	✓	–**	✓	✓	✓
	Diagnostic centers	–	–	✓	✓	–	–
	Research centers	–	–	–	–	✓	✓

		Reg.7 [45, 46]	Reg.8 [4, 30, 47, 51, 52]	Reg.9 [20, 48]	Reg.10 [33, 49]	Total
Geographical coverage	Regional	–	–	–	–	1
	National	–	–	✓	✓	5
	International	✓	✓	–	–	4
Purposes	Clinical	✓	✓	✓	✓	9
	Research-based	–	✓	✓	✓	8
	Epidemiological-based	✓	–	–	✓	5
Data set	Demographics	✓	✓	✓	✓	9
	Clinical history	✓	✓	✓	✓	9
	Paraclinical measures	✓	✓	✓	✓	9
	Treatment	✓	✓	✓	✓	9
	Outcomes	✓	✓	✓	✓	9
	Biological samples	–	–	–	✓	4
	Patient evaluation (safety/drug efficacy)	–	✓	–	–	1
Data quality assessment strategy	Completion of data	–	–	–	–	1
	Physician’s participation in data quality control	–	✓	✓	–	2
	Following data quality guidelines	–	–	–	–	1
	Using of data quality indicators	–	–	–	✓	1
	Evaluating and auditing data quality	–	–	–	–	2
	Training courses on data quality	–	–	–	–	1
	Data errors tracking	–	–	–	–	1
	Real-time evaluation of data at the time of its entry	–	–	–	✓	1
Inclusion criteria	Atypical HUS	–	✓	✓	–	8
	Typical UHS	✓	–	–	–	2
	Children	✓	✓	✓	–	9
	All ages	✓	✓	–	–	7
Exclusion criteria	Atypical HUS	*	–	–	–	2
	Typical UHS	–	✓	–	–	3
	Adults	–	–	✓	–	2
Registrars	Physicians (nephrologists)	✓	✓	✓	✓	8
Data collection	Retrospective	✓	–	✓	–	5
	Prospective	–	✓	✓	✓	8
Data sources	Healthcare centers	✓	✓	✓	✓	9
	Diagnostic centers	–	–	–	–	2
	Research centers	–	–	–	–	2

*With negative *E. coli* test

**No further information was found regarding data sources of this registry

HUS: Hemolytic Uremic Syndrome; Reg: Registry

Table 2 shows that these 10 HUS registries were set up from 1989 to 2013, the oldest of which is in Oklahoma, USA. America and Europe each have 4 registries, and Australia and Asia each have one HUS registry. The geographical scope of the registries was diverse. Half of them were national (5 registries), 4 registries were also international. The followings are the similarities and differences among each of the registries, based on the features:

1. The registry purposes were diverse, including clinical purposes (to manage and improve patient care), research-based (to evaluate patients and better understand the disease, discovering unknown factors and identifying treatments and outcomes of the disease), and epidemiological goals (to determine outbreak of HUS). The majority of registries had clinical purposes (Registries 1, 2, 4–10) [20, 31, 34, 38, 39, 42, 45, 49, 51] followed by research-based registries (Registries 1–3, 5, 6, 8–10) [20, 31, 34, 38–40, 49, 51]. Five registries also had epidemiological goals (Registries 4–7, 10) [31, 39, 42, 45, 49]. Out of the 10 selected registries, only three followed all three types of purposes (Registries: 5, 6, 10) [31, 39, 49].
2. Except for one registry whose data was not found (Registry number 3) [40], the required data set in most of the registries [20, 31, 34, 38, 39, 42, 45, 49, 51] included demographic data, medical and family history, para-clinical and diagnostic measures, treatment and pharmacological data, complications, and short and long-term outcomes. Among these registries, one has added information about the effectiveness of treatment and patient safety (Registry number 8) [30], and four registries have considered biobank-related data (Registries 1, 2, 5, 10) [31, 34, 38, 49].
3. Data quality assessment strategies included the monthly evaluation of data quality regarding the completeness and acceptability of data, and data audit (Registries 4 and 6) [39, 42], the participation of physicians (pediatric nephrologists) in collecting data, editing, and correcting data errors (Registries 8 and 9) [20, 30], increasing the rate of data completion (Registry number 1) [34], following guidelines and data quality training (both, Registry number 6) [39], using specific data quality indicators (Registry number 10) [49], data errors tracking (Registry number 4) [42], and real-time evaluation of data at the time of its entry (Registry number 10) [49].
4. Inclusion or exclusion criteria of these registries were mainly defined based on the age of patients and the type of HUS (typical or atypical). Most registries included atypical HUS (Registries 1–6, 8, 9) [20, 31, 34, 38–40, 42, 51] and all patients without any age limitation (Registries 1–4, 6–8) [30, 34, 38–40, 42, 45]. Italian registry of HUS included both typical and atypical HUS (Registry number 4) [42]. The German HUS registry is also limited to the typical HUS (Registry number 7) [45]. Two registries exclude patients with the negative *E. coli* test (atypical HUS) (Registries 4 and 7) [42, 45].
5. In the most of compared registries (Registries 1, 3, 5–10) [20, 30, 31, 34, 39, 40, 45, 49], the sub-specialists (pediatric nephrologists) themselves act as registrars.
6. One registry collects data retrospectively (collecting data about previously-diagnosed patients) (Registry number 7) [45], four registries perform prospectively (collecting data to monitor the specific results and outcomes of the disease in the future) (Registries 3, 5, 8, 10) [30, 31, 40, 49], and four other registries applies two methods (Registries 1,2, 4, 9) [20, 34, 38, 42].
7. The data resources of 9 registries include health care centers (such as specialized pediatric nephrology hospitals) (Registries 1, 2, 4–10) [20, 30, 31, 34, 38, 39, 42, 45, 49]. Nephrology research centers (Registries 5 and 6) [31, 39] and diagnostic centers such as specialized nephrology laboratories (Registries 3 and 4) [40, 42] also provide information to some of the HUS registries.

## Discussion

RDRs can be used to increase the knowledge about rare diseases and provide the samples to conduct the studies on the treatments and the quality of care for rare diseases [30]. However, lack of an executive plan, up-to-date documented strategy, clear measurable purposes, and evaluation framework may result in the inconsistent registries [53]. We found that multiple HUS registries have different and distinguishable features which can be learned to develop other registries, as discussed below.

### Purposes of HUS registries

By the rapid increase in the implementation of RDRs, the purposes of these systems have also become very diverse, from the management of patient clinical data to epidemiological and research purposes [16]. The specific purposes of RDR usually include identifying patients in families (due to being hereditary of most of the rare diseases) and identifying the course and evolution of disease, specific risks and outcomes, and supporting research (especially in the genetic field) as well as evaluation of drugs and treatments. In general, due to the knowledge gap in this field, the scope and purposes of RDRs are often wider than the other registries [19].

The purposes of the most HUS registries include optimal management and treatment of patients [32, 35, 36, 54], genetic evaluations in patients with HUS [20, 31, 37–40, 43, 48], and collection of HUS epidemiological data in different populations [31, 32, 41–43]. Consequently, due to the rarity of HUS, it seems that considering all three types of clinical, epidemiological, and research purposes to implement these registries is necessary. Woodward, et al. [30], in their study suggested that the purposes of HUS registry should include increasing knowledge about the disease, measuring the quality of HUS care, evaluating drug safety and other treatments which addresses both clinical and research aspects. Due to the lack of knowledge in the field of HUS management, research needs, and the lack of patient samples for research on this disease, it is recommended that specialists implement HUS registries with three aspects of clinical, research, and epidemiological purposes and meet these goals by establishing national or international systems to cover a large population.

### Inclusion and exclusion criteria

Depending on the registry purposes, inclusion criteria can be very extensive or limited but often include demographic characteristics (e.g., patients’ age), diagnosis, treatment, laboratory tests, or diagnostic methods [55]. Most non-rare and common diseases have a clearer and more developed classification and diagnostic criteria than rare diseases (such as HUS) [19]. Therefore, it is very important to determine one or more specific inclusion criteria for RDRs, such as HUS registries because it should be possible to detect all differential diagnoses through these criteria and enter exactly HUS patients.

Due to Vesely et al. [36], the Oklahoma Registry includes any patients with HUS or TTP at any age who are candidates for PEX, and the registry only excludes children with STEC-HUS that received conservative treatments. This makes it easy to identify and record the majority of HUS patients in the Oklahoma population. On the one hand, such a criterion is proper for maximum coverage of HUS patients because various factors affect the course of the syndrome, (aHUS especially), such as the age of onset, the severity of complications, and response to treatment [38]. Therefore, these patients may show different manifestations and not be recognized using a specific criterion. On the other hand, this criterion may lead to the entry of non-HUS patients who are wrongly candidates for PEX. Therefore, many HUS registries consider typical or atypical types of HUS (due to the presence or absence of Shiga toxin-producing *E. coli* (STEC)) along with the patient's age (children, or all ages) as their inclusion criteria because these criteria can be easily checked and confirmed by diagnostic and laboratory tests. Therefore, due to the rarity of HUS and the scattered patients, and the need to implement multicenter registries, it is recommended to use accurate and confirmable inclusion criteria such as a triad of thrombocytopenia, microangiopathic hemolytic anemia, and acute renal injury, type of syndrome, and the patient's age for case finding and patient enrollment, so that its data can be comparable.

### Minimum data set

The data elements of a registry should be limited and based on the purposes for which the registry is implemented [25]. Data expansion usually occurs over time, but a useful way to select the mandatory data elements and optional ones (data elements that are useful but unnecessary). Thus, the minimum set of important data on the RDRs includes key patient data (such as demographic data), family history and disease-related data including history and course of the disease (and other clinical data such as medical history, para-clinical and diagnostic procedures, treatment plans and prescribed drugs, short-term and long-term side effects or outcomes), data on the prevalence and distribution of the disease [19]. Moreover, patient's status (e.g., alive, dead, loss to follow-up or opted-out), care pathway (date of the initial encounter to the specialized center), research-related data (e.g., agreement or consent for reuse of patient's data and receiving a biological sample for research purposes), and patient's disability in accordance with International Classification of Functioning and Disability (ICF) are also suggested by European Commission Joint Research Centre for RDRs [56].

Managers of HUS registries also need these data sets, which exist in current HUS registries. Some researchers, such as Metjian et al. [49], stated that biological samples-related data should also be recorded to facilitate future research in addition to the mentioned data sets. These data are needed to address research challenges and shortages of biological research samples, especially to identify the relationship among genetic factors (by storing DNA and RNA samples) and incidence of HUS. In this case, by connecting multiple registries to a centralized biorepository or biobank, more samples can be provided to understand the disease course and the outcomes of patients [19].

Furthermore, Licht et al. [4] stated that in their HUS registry, data on treatment efficacy and patient safety in response to the new drugs such as Ecu was added to the registry by comparing outcomes in different groups of patients. Moreover, this type of data is very effective to evaluate the effectiveness of various treatments of HUS. Therefore, the applicants for the HUS registry implementation are recommended to pay special attention to collect data related to the biological samples of patients (if necessary) and the safety and efficacy of drugs. Based on these findings, the proposed minimum data set for an HUS registry is presented in Table 3.

**Table 3:** Proposed MDS for HUS registries

Data group	Data elements
Demographic and current episode data	Patient identity: first name, last name, date and place of birth, place of residence, age, sex Other patient data: weight, height, body mass index, race Encounter summary such as final diagnosis, residual symptoms (e.g., neurologic, cardiac, renal, bleeding) Data related to the patient care center and the referring physicians and centers
Medical and family history	History of the HUS, risk factors and causes of the disease such as infections, systemic diseases, drugs used concomitantly with the disease, history of kidney transplantation, family history, time from diagnosis to patient registration, age of onset, co-morbidities (e.g. hypertension, malignancy, etc.),
Signs and symptoms (clinical/laboratory)	Clinical: diarrhea (bloody or non-bloody), vomiting, nausea, abdominal pain, fever, number of days of onset of symptoms at the time of admission, time course of the acute illness, headache or dizziness, high blood pressure, paleness, lethargy Laboratory (at admission and discharge): Hematology, biochemistry, immunology (such as ANA) tests, urine analysis (creatinine, urea, and blood levels in urine), urine culture, stool culture (to detect *E. coli*), genetic tests such as Complement factors (B, H, I), C3, C4, ADAMTS13, and direct antiglobulin test (direct Coombs)
Para-clinical measures	Types of imaging of different parts of the body (ultrasound, MRI, etc.) and results
Therapeutic and pharmaceutical data	Drugs: Ecu (number, the date of the first dose, and continuation of PEX after the start of Ecu), Rituximab, Antibiotics, Steroids, Anticoagulants, Coagulants, Immunoglobulins, Corticosteroids Therapeutic measures: Plasma injections, PEX (date of first PEX, number of PEX), serum creatinine
Complications	Complications of drugs, therapeutic or diagnostic measures (during hospitalization) Efficacy and safety data for treatments, for example, meningococcal infections, sepsis, other serious infections, and death after Ecu or other medications
Short and long-term outcomes	Severe hypertension, renal and neurological dysfunction (with severity), recurrence of the disease, Slurred speech, Personality changes, Visual impairment, Seizures, Coma, Length of hospital stay, Problems with concentration, memory, and fatigue, Depression, Systemic lupus erythematosus, Hypertension, Diabetes mellitus, Hemorrhage, Sepsis, Cognitive dysfunctions, Death (date and cause), Splenectomy, Hemodialysis, Peritoneal dialysis, Kidney or liver transplantation
Data about biological samples	Plasma, urine, DNA, and umbilical cord (with sample details, date of sample collection, type of treatment, and laboratory values of patients on the date of sample collection)

ADAMTS13: a disintegrin and metalloproteinase with a thrombospondin type 1 motif, member 13; ANA**:** Antinuclear antibody; DNA: Deoxyribonucleic Acid; *E. coli*: *Escherichia coli*; Ecu: Eculizumab; HUS: Hemolytic Uremic Syndrome; MRI: Magnetic Resonance Imaging; PEX: Plasma Exchange

### Data sources

If RDRs collect data from multiple data sources, the results of these systems will be more effective [57]. Furthermore, the best approach for implementing RDRs is a collaborative approach in which multinational and multi-institutional stakeholders combine resources [19, 22]. Because of the non-outpatient nature of HUS, most of its data sources are nephrology (or pediatric nephrology) centers and hospitals. As stated by Woodward et al. [30]. In the French aHUS registry, patients are also identified from French Reference Laboratory for registration, so laboratories may also be one of the data sources for the HUS registry [40]. However, laboratory centers alone are not complete sources for providing HUS data, because laboratories are not usually set up specifically to assess HUS or other pediatric nephrology diseases and do not have comprehensive information in this regard. Therefore, it seems that to develop HUS registries, communication with a set of healthcare centers, diagnostic and nephrology research centers is necessary. On the other hand, registries may need to integrate data from different sources [55]; therefore, the possibility of using the Electronic Health Record (EHR) is very helpful because it facilitates the process of case findings and completing patient records or follow-ups from different data sources.

### Data collection method

HUS registries apply various methods for data collection. To collect comprehensive data on the disease course, the data should be collected through the interview with patients and receiving their previous information or reviewing the medical records of patients diagnosed (before launching the registry (retrospective) and also by collecting data over time and tracking the change of their symptoms or conditions (prospective). In their study, Kielstein et al. [45] stated that the data were collected retrospectively in the German HUS registry. In the other HUS registry, most data collection was prospective with a focus on data collection during patient visits. However, the global aHUS registry collects data both retrospectively and prospectively from pediatric nephrology centers [30], which maximizes the comprehensiveness of data related to before and after the disease.

Retrospective data collection can lead to missing data related to etiology, underlying diseases, family history of HUS, outcomes, and follow-ups because patient information may not be available before the registry is set up. On the other hand, just being prospective also prevents previously diagnosed patients from being registered, and due to the rarity of the HUS, this leads to the loss of many cases. Therefore, HUS researchers who intend to set up a registry are recommended to use both retrospective and prospective methods, considering the feasibility of data collection and prioritization of key variables for data collection from existing sources, current format, and limitations of information sources [58].

### Registry data quality control program

Assuring data quality is one of the most important features to design and maintain rare disease registries [22]. Moreover, HUS registries should develop a specific program and framework to evaluate and assure data quality.

Licht et al. [4] indicate physicians' increased participation in collecting, editing and correcting data errors to increase data quality and optimize the results of global aHUS registry. Another HUS registry applies the monthly evaluation of data quality due to the acceptability, completeness, and compatibility with the main data of patients [41, 42], and uses these information for prevention, identification, and correction of data errors regularly. Therefore, managers of HUS registries are advised to use a combination of methods to ensure data quality.

One of these methods is using coded data and classification systems, especially the use of Orphanet classification, definitions for recording or reporting particular data items due to internationally agreed-upon guidelines (especially in multi-center registries). Other methods also include determining the quality indicators [59, 60], such as determining the percentage of missed data [61, 62], comparing re-abstracted data with the main data from the data source [61], auditing the case-finding process, and case reporting [63] in registry centers over time, data audit with continuous feedback [59, 64] by data quality supervisors, data error correction at data entry, automatic data validation [64, 65] and warnings about mandatory data elements [65, 66].

Since HUS registries are mainly multicenter, setting up a data usage agreement between participant centers and countries will help determine the ownership of data and result in more participation and registration of HUS cases.

### Registrars

Because of this disease's sub-specialization nature, pediatric nephrologists act as a registrar in most of the existing registries. Woodward et al. [30] noted that in all HUS registry participant centers, pediatric nephrologists are responsible to enter the information of patients into the registry system because it is better to collect and enter data by personnel who are directly involved in the treatment and diagnosis of this disease [45]. Since physicians and specialists are busy, enforcing them to enter data is very challenging [29]. Therefore, to facilitate this task, it is recommended that the interoperability between the registry software and other information systems such as electronic health record systems (EHRs) [67, 68] should be considered to import all types of data from these systems to the registry software. Furthermore, issuing data quality certificates can motivate physicians to participate in a registry system to be increased [65].

**Fig. 1:**
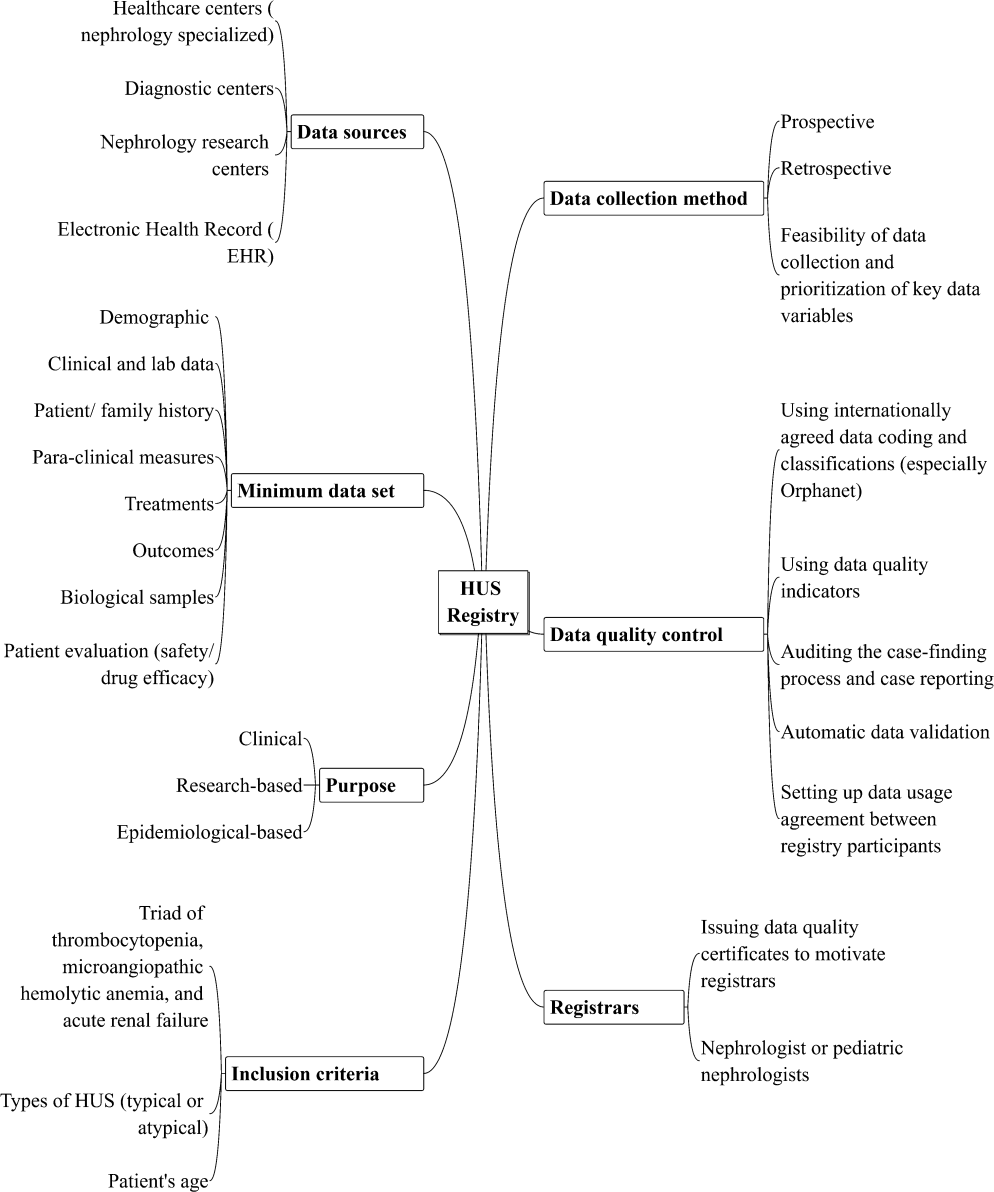
The suggested framework for implementing HUS registries

Due to the findings of study, the learning framework to implement HUS registries is presented in Fig. 1. This figure highlights the most important features to implement HUS registry, including purpose formulation, accurate inclusion/exclusion criteria, proper minimum data set, data sources, appropriate methods for data collection, and data quality control, as well as hiring knowledgeable registrars.

### Limitations

This study has some limitations. The comparison of registries was limited to the resources available. Some of the required information about the registries was incomplete. In these cases, we contacted their managers; however, this information was not completely obtained.

## Conclusion

This comparative study on selected HUS registries and their similarities and differences indicates that to develop and implement an HUS registry, a set of important features should be considered: 1. Formulation of comprehensive purposes on all three aspects of clinical, research, and epidemiology of HUS; 2. Developing appropriate inclusion criteria such as HUS diagnostic triad, determining typical or atypical HUS, and patient's age, 3. Developing an appropriate MDS; 4. Communication with different data sources, including healthcare centers (hospitals), diagnostic or nephrology research centers; 5. Data quality assurance uses various methods such as using agreed international coding and classification systems, using data quality evaluation indicators, case finding auditing, and data validation by the quality control supervisor or automatically with continuous feedback; and 7. Increased participation of nephrologists to enter data. By identifying and applying these features, managers and researchers can have more successful planning for implementing an HUS registry.

## Supplementary Information

**Additional file 1**. Detailed features of selected HUS registries.

## Data Availability

Data sharing is not applicable to this article as no datasets were generated or analyzed during the current study.

## Abbreviations

ADAMTS13: A Disintegrin and Metalloproteinase with a Tthrombospondin type 1 motif, member 13
aHUS: Atypical HUS
ANA: Antinuclear Antibody
ASFA: American Society for Apheresis
DNA: Deoxyribonucleic Acid
*E. coli*: *Escherichia coli*
Ecu: Eculizumab
EHR: Electronic Health Record
EHEC: Enterohemorrhagic *E. coli*
HUS: Hemolytic uremic syndrome
ICF: International Classification of Functioning and Disability
MDS: Minimum Data Set
MRIMRI: Magnetic Resonance Imaging
NA: Not Available information
NIH: National Institute of Health
PEX: Plasma Exchange
RDRs: Rare Disease Registries
Reg: Registry
STEC-HUS: Shiga toxin-producing *E. coli* HUS
TMA: Thrombotic Microangiopathy
TPE: Therapeutic Plasma Exchange
TRNA: TMA Registry of North America
TTP: Thrombotic Thrombocytopenic Purpura
