# Supplementary material for: Lessons learned from hemolytic uremic syndrome registries: recommendations for implementation
Source: Orphanet J Rare Dis. 2021 May 25;16:240. doi: 10.1186/s13023-021-01871-9 (PMC8146148; doi:10.1186/s13023-021-01871-9)
Supplement: Supplementary file 1 — Additional file 1. Detailed features of selected HUS registries. [file 13023_2021_1871_MOESM1_ESM.docx]

**Additional file 1**

List of included registries:

Registry 1: Oklahoma TTP-HUS Registry

Registry 2: International Registry of recurrent and familial Hemolytic Uremic Syndrome/Thrombotic Thrombocytopenic Purpura

Registry 3: French Registry of atypical Hemolytic Uremic Syndrome (aHUS) in children

Registry 4: Italian Registry of Hemolytic Uremic Syndrome

Registry 5: International Registry and biorepository for TMA (Thrombotic microangiopathy)

Registry 6: Thrombotic Thrombocytopenia Purpura / Thrombotic Microangiopathies Registry (TTP/TMA)

Registry 7: German STEC-HUS Registry

Registry 8: Atypical Hemolytic-Uremic Syndrome (aHUS) Registry

Registry 9: Turkish pediatric atypical hemolytic uremic syndrome Registry

Registry 10: TMA Registry of North America (TRNA)

Table 1 - Features of selected HUS registries

| Registry codes | -Registry name  -Year  -Country  -Geographical coverage | Purposes | Minimum data set | Data quality assurance | Inclusion and exclusion criteria | -Registrars  -Data collection method  -Data sources |
| --- | --- | --- | --- | --- | --- | --- |
| Registry 1 | - Oklahoma TTP-HUS Registry  - 1989  - US  - Regional **[34-36, 50]** | - Improving the care and treatment of patients with a diagnosis of HUS or TTP  - Supporting patients, and collecting information about patients’ evaluation and management for community physicians, and developing a resource for research and educational programs | - Demographic data (age, sex, weight, body mass index, race)  - Clinical features of patients such as thrombocytopenia and microangiopathic hemolytic anemia, neurological abnormalities with its severity, renal dysfunction, and its severity from acute renal failure to its normal function  -The presence or absence of fever  - Laboratory information, especially haematology and measurement of ADAMTS13 activity  - Complications, and long-term outcomes of the disease such as relapse, depression, systemic lupus erythematosus, hypertension, diabetes mellitus, and cognitive function outcomes (problems with concentration, memory and fatigue) | - Completion of documents and records of all patients  - Completion of entering data about patients  - Developing data collection forms  - Defining data  - Developing a comprehensive database | - Inclusion criteria: All consecutive patients with a diagnosis of TTP or HUS that are referred to the Oklahoma Blood Institute for which plasma exchange is requested, Obtaining informed consent  - Exclusion criteria: Without exclusion criteria (except for children who are not usually treated with plasma exchange and have STEC-HUS who only need supportive care.) | - NA  - Retrospective and prospective  - 11 hospitals in Oklahoma City region and other hospitals in surrounding communities |
| Registry 2 | - International Registry of recurrent and familial hemolytic uremic syndrome/thrombotic thrombocytopenic purpura  - 1996  - Italy  - International **[37-39]** | - Collection of clinical data and biological samples from patients and their families.  - Studying of genetic and biochemical abnormalities of HUS / PTT  - Providing information about the disease to health professionals and families  - Improving the possibilities of treatment and finding innovative therapeutic approaches. | - Demographic  -Referral centers and physicians  - History of disease and risk factors and disease triggers  - Early signs of disease and clinical manifestations  - Complications  - Treatments and drugs  - Laboratory data at admission and discharge from the hospital such as stool culture, etc.  -Outcomes such as peritoneal dialysis and hemodialysis  -Information on collected biological samples such as umbilical cord samples | NA | - Inclusion criteria: Patients with recurrent and familial HUS / TTP, ppatients’ age between 1 to 80 years  - Exclusion criteria: NA | - NA  - Retrospective and prospective collection of clinical data and biological samples through an international biobank network  -180 national and international children's and adult medical centers |

Table 1 (continue)

| Registry codes | -Registry name  -Year  -Country  -Geographical coverage | Purposes | Minimum data set | Data quality assurance | Inclusion and exclusion criteria | -Registrars  -Data collection method  -Data sources |
| --- | --- | --- | --- | --- | --- | --- |
| Registry 3 | - French registry of atypical Hemolytic Uremic Syndrome (aHUS) in children  - 2005  - France  - National **[40]** | Identification of phenotype-genotype correlation in children with atypical HUS in France | NA | NA | -Inclusion criteria: Patients with aHUS in children and sometimes adults  -Exclusion criteria: Patients with HUS with a bacterial infection | - Physicians  - At first, both retrospectively and prospective but then only prospective  - French reference laboratory for complement |
| Registry 4 | - Italian registry of hemolytic-uremic syndrome  - 2005  - Italy  - National **[41, 42]** | - Continuous collection of epidemiological and clinical information of patients with the typical or atypical HUS  - Improving clinical care and promoting basic research in the field of HUS  - Establishing cooperation with other registries and HUS research centers, both nationally and internationally  - Promoting the surveillance of HUS in Italy by collaborating in veterinary public health activities aimed at preventing Shiga toxin-producing E.coli (STEC) | - Demographic data such as the age of onset, place of residence, gender  - Information on disease status and potential risk factors  - Signs and symptoms at the onset of the disease  - Clinical and laboratory signs at the time of admission, during hospitalization and at the time of discharge (date and cause of death), and regular follow-ups  - Regular diagnostic information about Escherichia coli infection  - Epidemiological information on exposure to E.coli producing Shiga toxin | - Monthly data quality control by checking the level of completeness and acceptability of the data  - Sending a request to correct data errors to pediatric nephrology centers and follow up on correcting unacceptable data in case of data mismatch | - Inclusion criteria: Patients with typical HUS (with or without diarrhea) and aHUS in children and adults  - Exclusion criteria: Patients suspected of having aHUS with a negative diagnosis of STEC | - NA  - Retrospective and prospective data collection through a web-based system  - Pediatric nephrology centers in Italy and diagnostic laboratories and national reference laboratory for the diagnosis of E. coli |

Table 1 (continue)

| Registry codes | -Registry name  -Year  -Country  -Geographical coverage | Purposes | Minimum data set | Data quality assurance | Inclusion and exclusion criteria | -Registrars  -Data collection method  -Data sources |
| --- | --- | --- | --- | --- | --- | --- |
| Registry 5 | - International registry and biorepository for TMA (Thrombotic microangiopathy)  - 2007  - US  - International **[31, 43]** | -Determination of epidemiological information and outcomes of various diseases of TMA  - Determining the genetic causes of TMA  -Initiation of clinical trials for this group of diseases | - Demographic data  - Clinical data such as symptoms, diagnosis, laboratory data, treatment and outcomes  - Data from biological samples (such as plasma, urine, and DNA) | NA | - Inclusion criteria: Children from six months to 18 years of age with TMA conditions include: 1. severe STEC-HUS, 2. familial and non-familial aHUS, or 3. TTP.  - Exclusion criteria: This registry does not have exclusion criteria.. | - Physicians  - Prospective  - 8 children hospitals, research centers, and universities |
| Registry 6 | - Thrombotic Thrombocytopenia Purpura / Thrombotic microangiopathies registry (TTP/TMA)  - 2011  - Australia  - National **[32, 44]** | - Accurate definition of the incidence, natural history and clinical outcome of TTP and other TMAs  - Providing information on the range of therapeutic strategies being employed in the treatment of TMA patients  - Discovering the factors affecting clinical outcomes  -Optimal patient management | - Demographic data  - Diagnoses, clinical and laboratory data  - Imaging results,  -Therapy  Disease complications (such as Blood transfusion)  - Clinical outcomes | - Performing audits and data quality control  - Following guidelines to ensure repeatability, accuracy, consistency, and validity of data  - Planning and holding training courses and workshops on data quality control | - Inclusion criteria: all patients with TMA at any age (including TTP and aHUS)  - Exclusion criteria: NA | - Physicians  - NA  - 37 medical universities and research centers |

Table 1 (continue)

| Registry codes | -Registry name  -Year  -Country  -Geographical coverage | Purposes | Minimum data set | Data quality assurance | Inclusion and exclusion criteria | -Registrars  -Data collection method  -Data sources |
| --- | --- | --- | --- | --- | --- | --- |
| Registry 7 | - German STEC-HUS registry  - 2011  - Germany  - International **[45, 46]** | - Analysis of essential epidemiological and laboratory data, as well as clinical information about patients with HUS originating from E. coli producing Shiga toxin from hospital admission to discharge, and planning to prevent the spread of infection in the population.  - Helping to better manage the disease | - Demographic data such as gender, age, height, weight, and body mass index  - Clinical symptoms such as high blood pressure, body temperature, nausea, diarrhea, vomiting, number of days of onset of symptoms at admission, time course of the acute illness, headache, confusion  - Laboratory findings such as blood and urine analysis, presence of Enterohemorrhagic E. coli (EHEC) in stool sample  - Kidney function such as creatinine and urea levels  - Neurological symptoms such as headache or dizziness  - Treatment: Therapeutic plasma exchange (TPE), date of first TPE, number of TPE, steroids anticoagulation, Eculizumab (Ecu), the date of first Ecu, number of Ecu and continuation of TPE after the start of Ecu, serum creatinine and etc.  -Outcomes : Slurred speech, personality changes, visual impairment, seizures, coma, length of hospital stay, dialysis and neurological symptoms as well as overall mortality | Participation of physicians in entering and editing data periodically and collecting complete data | - Inclusion criteria: Patients with typical HUS, male and female, with a positive test for E. coli at any age.  - Exclusion criteria: Patients with a negative test for E.coli | - Nephrologists  - Collection of retrospective inpatient data  - 84 hospitals in Germany, Sweden and the Netherlands |

Table 1 (continue)

| Registry codes | -Registry name  -Year  -Country  -Geographical coverage | Purposes | Minimum data set | Data quality assurance | Inclusion and exclusion criteria | -Registrars  -Data collection method  -Data sources |
| --- | --- | --- | --- | --- | --- | --- |
| Registry 8 | - Atypical Hemolytic-Uremic Syndrome (aHUS) registry  - 2012  - US  -International  **[4, 30, 47, 51, 52]** | -Developing a global database of patients with atypical HUS to increase disease awareness by disseminating and analysing registry data  - Evaluating the long-term effects of the disease, including clinical outcomes and complications such as TMA complications and mortality in atypical HUS patients treated with Ecu or other treatments.  - Evaluation of safety and effectiveness of Ecu fulfilling post marketing regulatory requirements, and information on disease progression in patients. | - Demographic data, including age at diagnosis  - Medical history such as kidney transplant history  - Family history, symptoms, and duration of disease from diagnosis to patient registration  -Laboratory results (including genetic testing)  - Data on treatments such as plasma exchange and concomitant medications  - TMA complications  - Information on disease outcome and treatment effectiveness and safety outcomes | Participation of physicians in entering and editing data periodically and collecting complete data | - Inclusion criteria: Male or female patients of any age, including minors diagnosed with aHUS, regardless of treatment or management, patients with or without a specific pathogenic complement or causative antibody anti-complement, mandatory written and informed consent, disease cases with ADAMTS13 activity more than 5%  - Exclusion criteria: HUS due to Shiga toxin only, lack of informed written consent, disease cases with ADAMTS13 activity less than or equal to 5% | - Physician and researchers via online connection  - Prospective data collection over six-month periods based on a specific protocol  - 290 centres set up in 19 countries |
| Registry 9 | - Turkish pediatric atypical hemolytic uremic syndrome registry  - 2013  - Turkey  - National **[20, 48]** | Assessing clinical and genetic characteristics, treatment modalities, associated extrarenal findings, and clinical outcomes of patients during their initial hospital admission and evaluation of their long-term prognosis according to disease management strategies. | - Demographic characteristics  - Medical history and disease, clinical features at the onset of the disease (physical examination findings and laboratory data),  -Genetic results,  - Treatments performed in the acute stage (plasma injections, plasma exchange, Ecu, hemodialysis, peritoneal dialysis, and continuous renal replacement therapy)  - Kidney function, blood, protein in the urine, hypertension, serum creatinine level, hemoglobin level, platelet count, the status of the patient being discharged  - Side effects of drugs  -Follow-up data (every 3 months) including hematological and renal function parameters, current treatment methods and recurrence of the disease (after the last visit) | Editing and controlling the quality of the data by the experts in charge of each center at any time by connecting online to the registry | - Inclusion criteria: Children with symptoms of microangiopathic hemolytic anemia, Coombs negative test, thrombocytopenia and acute renal failure, patients under 18 years, and patients with typical HUS (caused by E. coli infection)  - Exclusion criteria: Adult patients even if they were first infected in childhood. | - Pediatric nephrologists  -Retrospective and prospective  - 26 pediatric nephrology hospitals |

Table 1 (continue)

| Registry codes | -Registry name  -Year  -country  -geographical coverage | Purposes | Minimum data set | Data quality assurance | Inclusion and exclusion criteria | -Registrars  -Data collection method  -Data sources |
| --- | --- | --- | --- | --- | --- | --- |
| Registry 10 | - TMA Registry of North America (TRNA)  - 2013  - US  - National **[33, 49]** | -Designing and developing a registry program to define best practices for diagnosis, treatment, and management of TMA  - Creating a multicenter clinical data set of patients to categorize TMA subsets  - Developing a multi-institutional platform for conducting observational and interventional TMA clinical trials  - Establishment of a national biorepository of samples from patients with TMA to facilitate future studies | - Demographic (age, gender, race) and epidemiological data  - Clinical history and medical co-morbidities (e.g. hypertension, malignancy, etc.) TMA presentation such as fevers, cardiac, hematologic symptoms  - Laboratory data (e.g. WBC, hemoglobin, hematocrit, platelet count, basic metabolic panel LDH, haptoglobin, schistocytes, direct antiglobulin test (direct Coombs) ADAMTS13 activity) at the time of diagnosis of TMA  - Treatments (e.g. TPE procedural details), with an emphasis on details and complications in the treatment process  - Clinical outcomes  - Data on patient samples collected  Episode summary such as final diagnosis, residual symptoms (e.g., neurologic, cardiac, renal, bleeding)  -Medications given (e.g., steroids, Rituximab, Ecluziumab) | - Using data quality indicators  - Online and real-time analysis of data in the web-based database of the registry | NA | - Physicians  - Prospective  - Medical centers of universities |

ADAMTS13: A Disintegrin and Metalloproteinase with a Tthrombospondin type 1 motif, member 13

aHUS: Atypical HUS

DNA: Deoxyribonucleic Acid

E. coli*:* Escherichia coli

Ecu: Eculizumab

EHEC: Enterohemorrhagic E. coli

HUS*:* Hemolytic Uremic Syndrome

NA: Not Available information

STEC-HUS: Shiga toxin-producing E. coli HUS

TMA*:* Thrombotic Microangiopathy

TPE*:* Therapeutic Plasma Exchange

TRNA: TMA Registry of North America

TTP: Thrombotic Thrombocytopenic Purpura
